# Bibliography

**Published:** 1892-04

**Authors:** 


Catching's Compendium of Practical Dentistry for 1891. By B. H. Catching, D.D.S., Editor and Publisher, Atlanta, Ga.

The second issue of this valuable work of reference is before us for 1891. It is gratifying to note that the indefatigable editor has improved on the first volume and has removed some of the objections apparent in that of 1890. If this work is continued from year to year it will be invaluable, not only to the busy practitioner, but to the writers of papers, who will be saved many hours of tedious labor in hunting for necessary facts.

The labor of condensation here exhibited has been great, and will, we fear, hardly be appreciated by the general reader. It must be said that, upon the whole, it has been well done. Each paragraph has the name of the journal from which the selection has been made, with the month of issue.

The subjects are classified under Operative Dentistry, Prosthetic Dentistry, Crown-, Bridge-, and Inlay-Work, Orthodontia, Dental Medicine, Oral Surgery, Miscellaneous, Synopsis of State Laws, List of Dental Journals of the World, together with Books and Pamphlets pertaining to Dentistry published during the year, making a book of two hundred and forty-one pages.

We find one line on page twenty-three for which the editor is responsible, and which, embalmed in the pages of a book supposed to represent the best thought of the year, seems out of place.

Dr. Catching says: "Sensitive Dentine.—Chloride of zinc is still one of the best remedies." In view of all the facts, this statement appears to be misleading, and may be the means of doing much harm.

Notice is especially made to this from the fact that a recent paragraph has called attention to the value of oxychloride of zinc for the same purpose. These views have probably been stimulated by the idea promulgated by a prominent writer that coagulation of albumen means a cessation of the destructive effect of the escharotic. No greater error, in our judgment, has ever been sought to be established. Chloride of zinc, from its great affinity for moisture, will continue the coagulation of albumen until it is thoroughly satisfied, whether this be in the tubuli or pulp-canals. Simple experiments with egg albumen will macroscopically demonstrate this, and the microscope will establish the fact for the smaller conduits in dentine. This being true, nothing worse could be placed in a cavity for a reduction of sensitive tissue, for sooner or later devitalization of the pulp is almost sure to follow.

We hope for an extended sale for this book, that the editor and publisher may be encouraged to continue it from year to year.

Manual of Instruction on Hard Soldering. By Harvey Rowell. E. & F. N. Spon & Co., New York and London. 1891.

The value of a book properly prepared on soldering of metals must depend on its meeting the needs of all classes of workers. This is a small work of fifty-four pages, but it covers much of great practical value.

Dentistry needs, just at this time, a book upon this subject. The long interregnum between the period of metal bases and that of bridge-work gave a class of men with little or no knowledge of metal-work or the experience so necessary to make good soldering. The result is that many are incapable of making even a gold crown properly.

The rules given in this book satisfactorily meet the requirements of small work, but will not aid the dentist in more complicated cases. This kind of soldering is peculiar to our profession and demands special instruction.

If this book should reach a second edition, we would advise the author to secure the services of a practical man to prepare this, in addition to that already given. This done, it would be a book necessary for every dentist. As it is, it will be found an exceedingly useful companion in the daily work of the laboratory.

LIPPINCOTT'S for April opens with a novel by Rose Nouchette Carey entitled "But Men Must Work." A chatty article by Melville Phillips is "The Literary Editor." "Nihilism and the Famine," by the Countess Norraikow, is a very timely paper and an exceedingly interesting account of the men and women prominent in this, the most remarkable effort for political freedom witnessed in the present century. The portraits given of the principal actors in the Nihilistic movement add greatly to the interest of the article.

The number contains other valuable contributions, and fully maintains its high character.

